# Age‐related changes in reticulospinal contributions to anticipatory postural adjustments between back extensors and abdominal muscles

**DOI:** 10.1113/EP091698

**Published:** 2024-05-15

**Authors:** Shin‐Yi Chiou, Catherine Unwin, Alice Lilley

**Affiliations:** ^1^ School of Sport, Exercise and Rehabilitation Sciences, College of Life and Environmental Sciences University of Birmingham Birmingham UK; ^2^ Sandwell and West Birmingham NHS Trust Treatment centre, City Hospital Birmingham UK; ^3^ Musculoskeletal Outpatients Department Queen's Hospital Burton Burton‐On‐Trent Staffordshire UK

**Keywords:** ageing, descending pathways, postural control, StartReact, subcortical structures, trunk

## Abstract

Anticipatory postural adjustments (APAs) give feedforward postural control of the trunk, but they are delayed with ageing, affecting balance and mobility in older individuals. The reticulospinal tract contributes to postural control of the trunk; however, the extent to which age‐related changes affect the reticulospinal contributions to APAs of the trunk remains unknown in humans. Here, we tested the hypothesis that a startling acoustic sound, which activates the reticulospinal tract, improves delayed APAs in older individuals. Twenty‐two old (75 ± 6 years) and 20 healthy young adults (21 ± 4 years) performed a self‐initiated fast bilateral shoulder flexion or shoulder extension task in response to visual, visual and auditory (80 dB), or visual and startling (115 dB) cues. Electromyography (EMG) was recorded from bilateral anterior deltoid (AD) and erector spinae (ES) during shoulder flexion and from bilateral posterior deltoid (PD) and rectus abdominis (RA) during shoulder extension. EMG onset of all muscles shortened during the startling cue in both age groups, suggesting a non‐specific modulation of the reticulospinal tract on prime movers (AD or PD) and non‐prime movers (ES or RA). Interestingly, APAs of the ES were accelerated in older participants to a similar degree as in younger participants during the startling cue. Conversely, APAs of the RA were not influenced by the startling cue in older participants. Our results suggest differential effects of ageing on functional contributions of the reticulospinal tract to APAs between back extensors and abdominal muscles.

## Abstract (highlights)

- **What is the central question of this study?**
  Does the reticulospinal tract contribute to anticipatory postural adjustments (APAs) of trunk muscles, and what is the effect of ageing on the reticulospinal contributions to the APAs of the trunk?
- **What is the main finding and its importance?**
  Startling stimuli shortened APAs of the trunk extensors during shoulder flexion, but not the trunk flexors during shoulder extension, in older participants. However, quiet acoustic stimuli did not affect APA control of the trunk muscles. Our results suggest an increase in the reticulospinal input to the trunk extensors for anticipatory postural control with ageing.

## 1 INTRODUCTION

Balance deterioration is commonly seen with ageing and is associated with risks of falling in the ageing population (Cuevas‐Trisan, 2017). The ability to maintain balance in upright posture requires control of the centre of mass over the base of support (Hess et al., 2006). There are two mechanisms used by the central nervous system to maintain or restore balance and posture: anticipatory postural adjustments (APAs) and compensatory postural adjustments (CPAs). The APAs are defined as changes in the level of background activity of postural muscles that happen in a window of −100 ms and +50 ms with respect to a predictable postural perturbation (Belen'kii et al., 1967). Since the timing of the APAs is too fast to be a result of peripheral modulation, the APAs are considered to be pre‐planned and therefore a feedforward mechanism mediated, at least in part, by the cortex (Chiou et al., 2016, 2018). Research has shown delays in the onset of APAs in older adults during self‐initiated perturbations (i.e., fast unilateral or bilateral arm movements; Bleuse et al., 2006; Inglin & Woollacott, 1988; Rowland et al., 2021) and external perturbations (i.e., externally triggered perturbations; Aruin et al., 2015; Bugnariu & Sveistrup, 2006). The underlying mechanisms are likely related to a decline in physiological responses in several body systems with ageing.

It is known that the number of corticospinal fibres is decreased with ageing (Terao et al., 1994). Work using transcranial magnetic stimulation (TMS) has reported that the amplitudes of motor evoked potentials obtained in the erector spinae (ES) prior to the onset of a fast shoulder movement correlated with the onset of ES activity, so as to counterbalance the perturbation to the trunk induced by the shoulder movement in older adults (Rowland et al., 2021). This suggests that corticospinal excitability is able to reach a functional level prior to the movement onset, which is relevant to the onset of the APAs in older individuals. In addition to the corticospinal tract, the reticulospinal tract has been suggested for postural control of axial muscles (Prentice & Drew, 2001; Schepens & Drew, 2004). Research has shown age‐related changes in the reticulospinal modulation in motor function. For example, older adults who maintained or enhanced their reticulospinal function showed greater grip strength than their peers (Maitland & Baker, 2021). Additionally, neurophysiological studies reported increased short‐latency responses in leg muscles elicited by mechanical taps to the trunk in older adults, suggesting an enhanced reticulospinal output in the context of postural perturbations in old age (Colebatch & Govender, 2018; Jeyakumar et al., 2018). So far, the contributions of the reticulospinal tract to control of APAs and the effect of ageing on the reticulospinal contributions to the APAs remain unknown.

Previous studies reported that APAs of trunk muscles occurring prior to limb movements were associated with direction of the movements. Specifically, activity of the trunk muscles anticipates reactive forces produced by the limb movements (Hodges & Richardson, 1997a; Hodges & Richardson, 1997b). For example, anticipatory activity of the erector spinae (ES) is often present in a fast shoulder flexion movement, whilst the activity of the rectus abdominis (RA) is usually observed in a shoulder extension movement. Prior work has shown that a decline in maximal strength of trunk flexors happened earlier than that of trunk extensors in older individuals (Sasaki et al., 2018), suggesting different effects of ageing on the ES and RA. Given the reticulospinal contributions to maximal motor outputs (Skarabot et al., 2022), we hypothesised a different contribution of the reticulospinal pathways to APAs of the ES and RA as well as a different effect of ageing on the reticulospinal contributions to APAs of the ES and RA.

In humans, the reticulospinal tract can be evaluated using an established method: the StartReact paradigm. The StartReact response occurs when an acoustic startling stimulus causes an early release of prepared movements (Valls‐Sole et al., 1999). Many studies have reported a clear shortening in voluntary reaction time under an acoustic startling stimulus compared with a normal acoustic stimulus or a visual stimulus (Baker & Perez, 2017; Dean & Baker, 2017). Evidence suggests that, at least for simple actions, detailed muscle movements can be stored and prepared in advance so they can be released quicker through limited cortical involvement when a startling stimulus is heard (Stevenson et al., 2014). The shortening in reaction time (StartReact response) may be related to activation of subcortical structures such as the reticular formation. Indeed, patients with dysfunction of the reticular formation were less affected by the startling cue in their reaction time (Nonnekes et al., 2014). To test our hypotheses, we examined APAs of the ES and RA during self‐initiated shoulder flexion and shoulder extension movement, respectively, in young and older adults using the StartReact paradigm.

## 2 METHODS

### 2.1 Participants

The study protocol was approved by the University of Birmingham STEM Research Ethics Committee (ERN_20‐1453) and conforms to the standards by the *Declaration of Helsinki*. All participants gave written informed consent. Twenty‐two older adults aged 65 years and above (75 ± 6 years; 14 females, 8 males) and 20 healthy young adults (21 ± 4 years; 17 females, 3 males) aged between 18 and 64 years were recruited. All participants were able to stand and walk independently without the use of a walking aid. Participants were excluded if they had any current or recent (within the last 6 months) musculoskeletal pain in the upper extremities or back (including low back pain and sciatica). They were also excluded if they had any falls or surgery in the last 6 months, had hearing loss and did not wear hearing aids, or suffered from any neurological conditions. Participants using hearing aids for hearing correction were instructed to wear them as prescribed.

### 2.2 Electromyography

Electromyography (EMG) was recorded bilaterally from the anterior deltoid (AD), posterior deltoid (PD), erector spinae (ES) and rectus abdominis (RA), and unilaterally from the sternocleidomastoid (SCM) muscle, with pairs of Ag–AgCl surface electrodes secured to the skin over the belly of each muscle (2 cm diameter, Almevan, Madrid, Spain). A ground electrode was positioned on the right superior iliac crest. The ES electrodes were placed 3 cm lateral from the midline of the spinous processes at the 12th thoracic vertebral level (T12). RA electrodes were placed 3 cm laterally of the umbilicus and 2 cm distally. Electrodes for PD and AD were placed in the middle of the muscle belly. All electrodes were placed with an inter‐electrode distance of 2 cm apart. The EMG signals were amplified (×1000) and filtered (10–1000 Hz) by a Digitimer Isolated Patient Amplifier System (Digitimer, Welwyn Garden City, UK), and then sampled at 2 kHz using a Micro1401‐4 data acquisition system and Signal software version 6.05 (Cambridge Electronic Design, Cambridge, UK).

### 2.3 Experimental procedures

**FIGURE 1:**
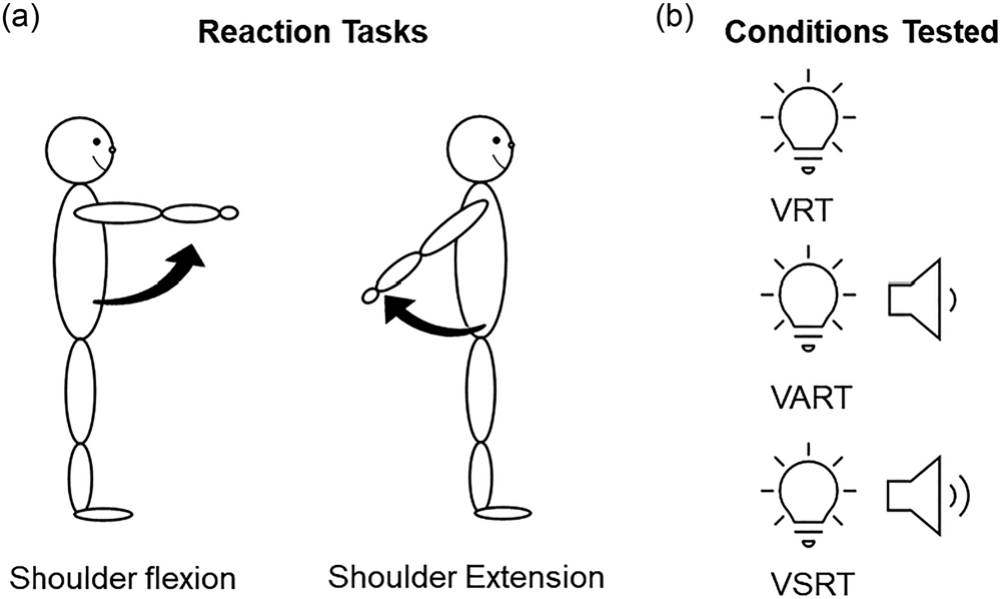
Experimental set‐up. (a) Schematic representation of the reaction tasks of shoulder flexion and shoulder extension. (b) Representation of the paradigm for evaluating the StartReact response in both young and older participants. A red LED comes on alone (visual reaction time condition, VRT), or in unison with an acoustic stimulus (80 dB; 500 Hz; 20 ms; visual acoustic reaction time condition, VART) or a startling acoustic stimulus (115 Db; 500 Hz; 20 ms; visual startle reaction time conditions, VSRT).

All participants were standing with feet at shoulder width and both arms relaxed by the sides, at 1 m in front of a red light‐emitting diode (LED) placed at eye level. The participants were instructed to perform shoulder flexion to 90° or shoulder extension to their maximal range of motion (45°−60°) due to anatomical constraints as fast as possible in response to the LED, which illuminated for 20 ms at a time (Figure 1a). All participants were shown how to perform the tasks by a researcher and given a familiarisation trial (see below for details) before the start of data collection. This simple reaction time paradigm was shown to elicit APAs of the ES and RA (Hodges & Richardson, 1997b; Hodges et al., 1999). Reaction time of each muscle was recorded using EMG and defined as visual reaction time (VRT). To examine the StartReact response, we used a previously tested paradigm (Fisher et al., 2013; Baker & Perez, 2017). Briefly, in some trials the LED was presented together with a quiet acoustic sound (80 dB; 500 Hz; 20 ms) or with a startling acoustic sound (115 dB; 500 Hz; 20 ms) (Figure 1b). The auditory stimulus was delivered by a speaker (150W 5‐Inch Active Pulse Audio (PA)/Monitor Speaker, UK) placed behind the participant's head. Decibel levels were confirmed using a RS Pro RS‐95 Sound level meter (35–130 dB, 8 kHz max, RS, Corby, UK) prior to testing. The startling sound evoked a clear startle in some young and older participants on the first presentation. Reaction times obtained from the quite acoustic conditions and the startling acoustic conditions were referred to as visual acoustic reaction time (VART) and visual startle reaction time (VSRT). Since an auditory stimulus reaches to the cortex earlier than a visual stimulus (Kemp, 1973), it is expected that auditory reaction time is faster than visual reaction time (Shelton & Kumar, 2010). Additionally, a startling stimulus is thought to activate additional descending pathways, such as the reticulospinal tract (Dean & Baker, 2017). Therefore, including VRT, VART and VSRT in the study paradigm allowed us to evaluate the effects of auditory stimuli (VRT vs. VART) and volume of the auditory stimuli (VART vs. VSRT) on the reaction time and APAs calculated from EMG. Prior to data collection, all participants underwent a familiarisation trial consisting of one time of each condition per task (Figure 1). During the data collection, participants were randomly allocated to complete the shoulder flexion task, followed by the shoulder extension task, or vice versa. In each task, 20 trials were recorded in each condition (VRT, VART, VSRT) in a randomised order with an interval between trials of 6–10 s. Breaks were given when needed.

Following completion of the tasks, participants performed three brief maximal voluntary contractions (MVCs; ∼3 s) with the trunk into extension (ES MVCs) or flexion (RA MVCs), separated by 60 s of rest to allow normalisation of EMG. For ES MVCs, participants lay prone on the plinth with straps securing the ankles and pelvis on the plinth and they were instructed to perform trunk extension. Resistance was applied against the scapulae for all contractions. For RA MVCs, participants were semi‐seated on the plinth with the backrest set to 45 degrees, with one strap securing the feet and the other across the chest to provide resistance; participants were instructed to flex their trunk. Verbal encouragement was given consistently throughout to ensure maximal effort was being obtained.

**FIGURE 2:**
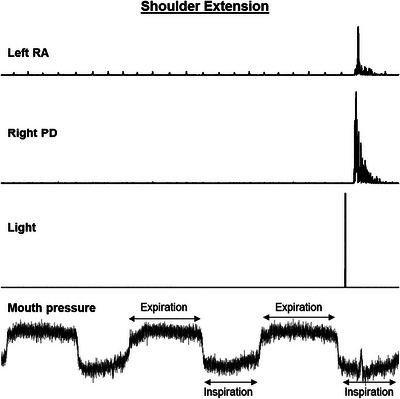
Recordings from a representative young participant showing rectified traces of left rectus abdominis (RA) and right posterior deltoid (PD) during the visual reaction time condition of shoulder extension. Heart beats are visible in the raw EMG traces of left RA. Positive and negative mouth pressure indicate expiration and inspiration, respectively. Note that the light is present during inspiration.

Furthermore, given that the RA can be activated during expiration of respiration to assist pushing the air out from the chest wall, we repeated the experiment in five young participants and triggered the stimulus when participants were in the inspiration phase by monitoring breath‐by‐breath pressure traces (Figure 2). Mouth pressure (113253, Hans Rudolph Inc., Shawnee, KS, USA) was recorded from a tube connected to a face mask (7450 V2, Hans Rudolph Inc.).

### 2.4 Data analysis

EMG data were firstly visually inspected to remove trials with failure to respond and poor signals. The onset time of the AD, ES PD, and RA muscles was measured manually trial‐by‐trial from the rectified EMG traces using Signal version 6.05. The onset was defined as a point where the mean rectified EMG exceeded three standard deviations calculated from the mean EMG level in a 50 ms window prior to the LED presentation (Chiou & Strutton, 2020; Hodges et al., 1999). EMG obtained from ES and RA during MVCs was rectified and calculated as mean EMG in a 500 ms window. APAs were calculated by subtracting EMG onset of AD from ES (i.e., ES minus AD) and EMG onset of PD from RA. Negative values indicated the postural muscles (ES, RA) were activated earlier than the prime movers (AD, PD).

### 2.5 Statistical analysis

SPSS Statistics version 28 (IMB Corp, Armonk, NY, USA) was used for statistical analysis. Normality was tested using the Shapiro–Wilk test. Sphericity was tested using the Mauchly test. When data were not normally distributed, non‐parametric tests were applied. When sphericity was violated (*P* < 0.05), the Greenhouse–Geisser statistic was used. Three‐way repeated‐measures ANOVA was used to determine the effect of Group (young vs. older), Condition (VRT, VART, VSRT), Muscle (shoulder flexion: AD, ES; shoulder extension: PD, RA) and their interaction on EMG onset, APAs and pre‐stimulus EMG in each task (shoulder flexion and shoulder extension). Additional repeated‐measures ANOVAs were used to examine an interaction between factors when needed. Moreover, repeated‐measures ANOVAs were applied to examine the effect of Group and Condition, and their interaction on mean EMG amplitudes during shoulder flexion and shoulder extension. *Post hoc* tests with Bonferroni corrections were applied if there was a significant effect. Furthermore, an independent Student's *t*‐test was used to test MVCs between groups. Furthermore, tests for two independent samples were used to test MVCs between groups, and two related samples were applied to compare MVCs between the ES and RA. Correlation analysis was used to determine a relationship between changes in APAs induced by the startling stimulus and MVCs if needed. Descriptive statistics was used for the subgroup analysis in the five participants with respiratory data. Statistical significance was set at *P* < 0.05. Data are presented as means ± SD in the text.

## 3 RESULTS

### 3.1 EMG

Data from 20 young and 22 older participants were included in the results. Mean rectified EMG amplitudes during ES MVC were greater in young participants (0.20 ± 0.08 mV) than in older participants (0.15 ± 0.12 mV; *Z* = −2.58; *P* = 0.01). On the contrary, EMG activity during RA MVC was not different between groups (young: 0.12 ± 0.08 mV; older: 0.11 ± 0.08 mV; *Z* = −0.40; *P* = 0.69). Furthermore, ES MVC was greater than RA MVC in both groups (young: *Z* = −3.14; *P* = 0.002; older: *Z* = −2.32; *P* = 0.02). Repeated‐measures ANOVA showed an effect of Muscle (*F*_1,40_ = 15.99, *P* < 0.001; η_p_^2^ = 0.29), but no effect of Condition (*F*_2,80_ = 0.49, *P* = 0.62) or an interaction of Muscle × Condition × Group (*F*_2,80_ = 0.37 *P* = 0.69) on mean rectified EMG activity measured prior to the stimulus presentation in the shoulder flexion task. Overall, ES EMG (0.01 ± 0.007 mV) was greater than AD EMG (0.006 ± 0.004 mV; *Z* = −4.70; *P* < 0.001) in standing. However, there was no effect of Muscle (*F*_1,40_ = 3.61, *P* = 0.07), Condition (*F*_2,80_ = 1.34, *P* = 0.27), or interaction of Muscle × Condition × Group (*F*_2,80_ = 1.73, *P* = 0.18) on mean rectified EMG activity prior to the stimulus presentation in the shoulder extension task, suggesting that EMG activity of the RA and PD was similar across all conditions in both groups. These indicated that the ES was activated prior to activity of the AD, whereas the RA and PD were activated at the same time in our cohort.

During shoulder flexion, results revealed an effect of Condition (AD: *F*_2,80_ = 28.06, *P* < 0.001; η_p_^2^ = 0.45; ES: *F*_2,80_ = 68.70, *P* < 0.001; η_p_^2^ = 0.63) but no interaction of Condition × Group (AD: *F*_2,80_ = 1.01, *P* = 0.37; ES: *F*_2,80_ = 1.51, *P* = 0.23) on EMG activity of AD and ES during shoulder flexion. EMG activity in the AD and ES was greater during VSRT than during VART (both *P* < 0.001). Additionally, EMG activity in the ES was greater during VART than during VRT (*P* = 0.002), whilst AD EMG was the same during VART and VRT (*P* = 0.26). Similar results were found during shoulder extension. There was an effect of Condition (PD: *F*_2,80_ = 34.36, *P* < 0.001; η_p_^2^ = 0.46; RA: *F*_2,80_ = 22.12, *P* < 0.001; η_p_^2^ = 0.36) but no interaction of Condition × Group (PD: *F*_2,80_ = 1.39, *P* = 0.26; RA: *F*_2,80_ = 0.81, *P* = 0.45) on EMG activity of PD and RA during shoulder flexion. EMG activity of the PD and RA was larger during VSRT than during VART (both *P* < 0.001). Moreover, both PD and RA EMG amplitudes were larger during VART than during VRT (both *P* < 0.001). Furthermore, EMG activity of the ES correlated with that of AD (*r* = 0.52; *P* < 0.001), and EMG activity of the RA also correlated with that of PD (*r* = 0.43; *P* = 0.004), indicating greater activity of the trunk muscles associated with greater activity of the shoulder muscles.

### 3.2 EMG onset

**FIGURE 3:**
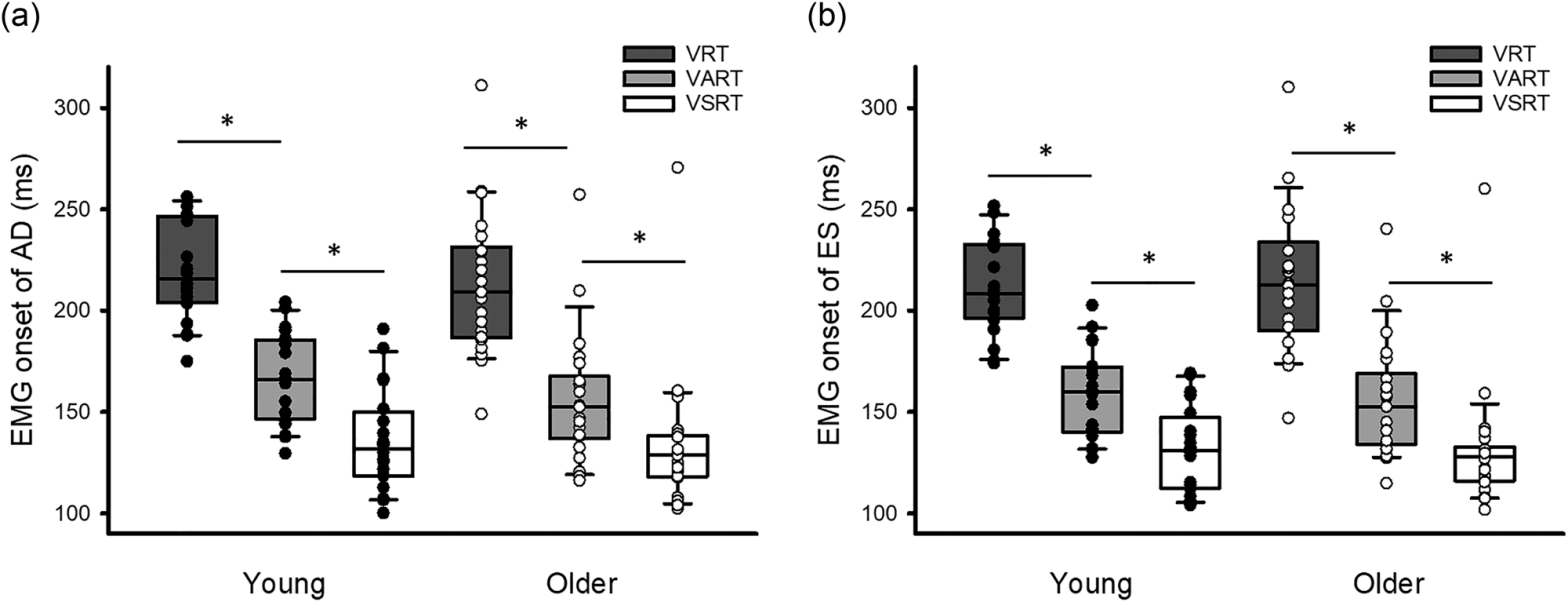
EMG onset during the shoulder flexion task. (a) Group data (young: *n* = 20; older: *n* = 22) showing electromyography (EMG) onset of anterior deltoid (AD) during visual, visual acoustic and visual startle reaction time conditions (VRT, VART and VSRT, respectively) in both groups. (b) Group data (young: *n* = 20; older: *n* = 22) showing EMG onset of erector spinae (ES) during the three conditions in both groups. The abscissa shows the EMG onset. The ordinate shows the group. Continuous lines indicate median values, and outer edges of the box represent the 25th and 75th percentiles. The upper and lower whiskers denote maximum and minimum values. Filled and open circles indicate individual data of young and older participants, respectively. *P < 0.05.

Data from all participants were included in the results. Repeated‐measures ANOVA revealed an effect of Condition (*F*_2,80_ = 440.09, *P* < 0.001; η_p_^2^ = 0.92), Muscle (*F*_1,40_ = 6.53, *P* = 0.014; η_p_^2^ = 0.14) and an interaction of Condition × Muscle × Group (*F*_2,80_ = 6.27, *P* = 0.003; η_p_^2^ = 0.14), but no effect of Group (*F*_1,40_ = 0.15, *P* = 0.71) on EMG onset during shoulder flexion. *Post hoc* paired *t*‐tests demonstrated that EMG onset of AD was shorter during VSRT (young: 135.27 ± 25.39 ms; older: 133.83 ± 34.11 ms) than during VART (young: 165.57 ± 23.21 ms; older: 156.35 ± 32.03 ms; *P* < 0.001). EMG onset of AD was also shorter during VART than VRT (both *P* < 0.001; Figure 3a). Similarly, EMG onset of ES during VSRT (young: 132.35 ± 20.42 ms; older: 131.60 ± 31.51 ms) was shorter than during VART (young: 160.73 ± 20.16 ms; older: 156.03 ± 28.96 ms; both *P *< 0.001). EMG onset of ES was also shorter during VART than VRT (young: 212.10 ± 24.47 ms; older: 211.85 ± 33.84 ms; both *P* < 0.001; Figure 3b).

**FIGURE 4:**
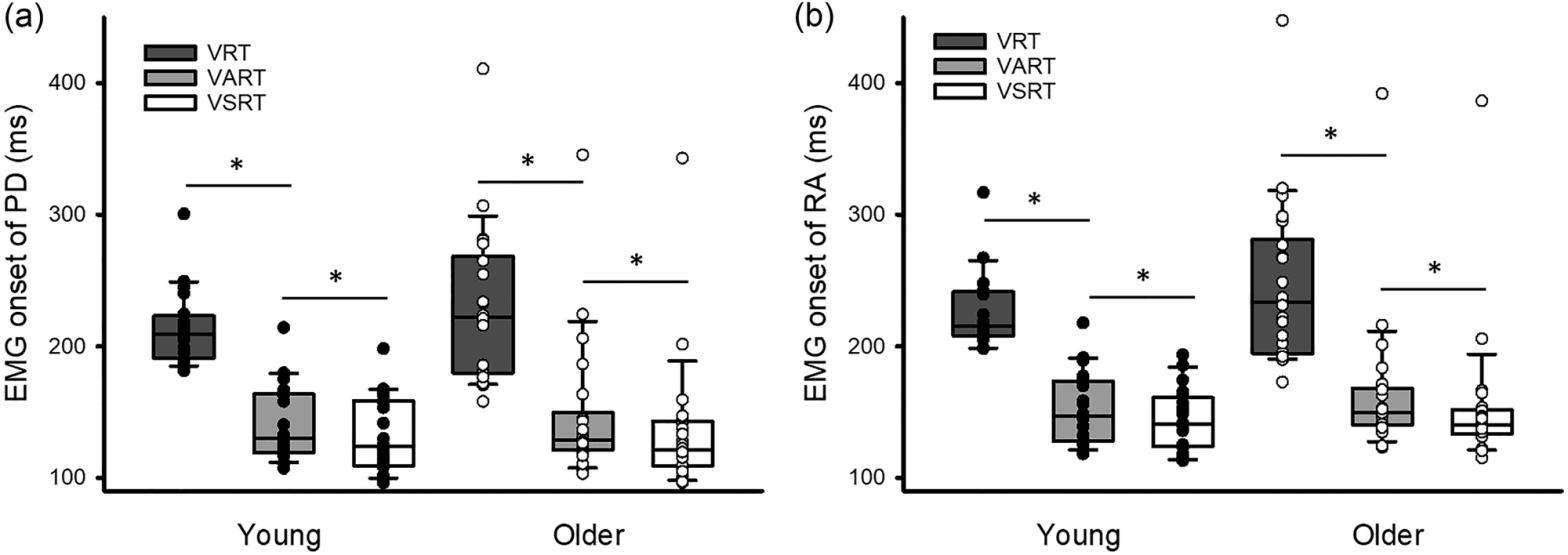
Electromyography (EMG) onset during the shoulder extension task. (a) Group data (young: *n* = 20; older: *n* = 22) showing EMG onset of posterior deltoid (PD) during the visual, visual acoustic and visual startle reaction time conditions (VRT, VART and VSRT, respectively) in both groups. (b) Group data (young: *n* = 20; older: *n* = 22) showing EMG onset of rectus abdominis (RA) during the three conditions in both groups. The abscissa shows the EMG onset. The ordinate shows the group. Continuous lines indicate median values, and outer edges of the box represent the 25th and 75th percentiles. The upper and lower whiskers denote maximum and minimum values. Filled and open circles indicate individual data of young and older participants, respectively. *P < 0.05 across conditions.

Furthermore, results showed an effect of Condition (*F*_2,80_ = 319.60, *P* < 0.001; η_p_^2^ = 0.89), and Muscle (*F*_1,40_ = 62.57, *P* < 0.001; η_p_^2^ = 0.61), but not interaction of Condition × Muscle × Group (*F*_2,80_ = 1.42, *P* = 0.25) on EMG onset during shoulder extension. *Post hoc* tests revealed that EMG onset of PD was earlier during VSRT (young: 132.80 ± 28.33 ms; older: 136.05 ± 51.93 ms) than during VART (young: 142.59 ± 28.56 ms; older: 147.76 ± 53.91 ms; *P* < 0.001), and was earlier during VART than VRT (young: 213.97 ± 28.25 ms; older: 231.46 ± 59.29 ms; *P* < 0.001; Figure 4a). Also, EMG onset of RA was shorter during VSRT (young: 145.19 ± 23.44 ms; older: 154.25 ± 55.17 ms) than during VART (young: 152.26 ± 27.30 ms; older: 165.09 ± 55.47 ms; *P* < 0.001) and was shorter during VART than VRT (young: 226.07 ± 28.60 ms; older: 246.60 ± 62.79 ms; *P* < 0.001; Figure 4b).

### 3.3 APA

**FIGURE 5:**
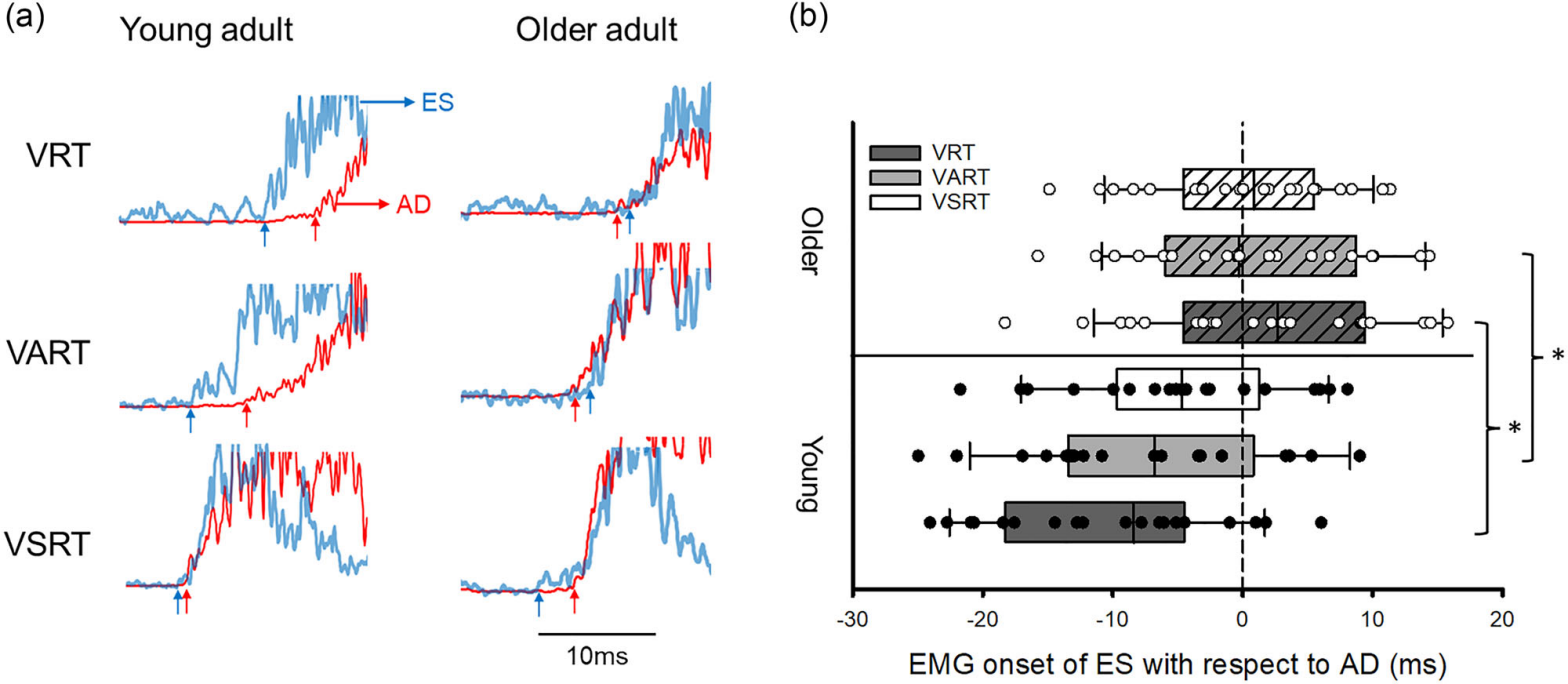
Anticipatory postural adjustments (APAs) during the shoulder flexion task. (a) Rectified electromyography (EMG) traces in representative young (left) and older participants (right) during the visual, visual acoustic and visual startle reaction time condition (VRT, VART and VSRT, respectively). EMG onset was measured during all conditions and arrows below each of the traces indicate the EMG onset in each of the conditions tested. (b) Group data (young: *n* = 20; older: *n* = 22) showing the difference in EMG onset between the erector spinae (ES) and anterior deltoid (AD). The abscissa shows the group. The ordinate shows the difference in EMG onset of ES to AD. Continuous lines indicate median values, and outer edges of the box represent the 25th and 75th percentiles. The upper and lower whiskers denote maximum and minimum values. Filled and open circles indicate individual data of young and older participants, respectively. *P < 0.05 comparisons between groups.

Older participants showed delayed APAs of the ES (*Z* = −3.55; *P* < 0.001; older: 3.07 ± 11.76 ms; young: −9.97 ± 8.80 ms) and RA (*Z* = −2.11; *P* = 0.035) during VRT in shoulder flexion and shoulder extension, respectively, compared with young participants. Furthermore, we examined the influence of the startling stimulus on APAs of the trunk. Figure 5a illustrates rectified EMG traces of AD and ES during VRT, VART and VSRT in a representative young participant and older participant. Note that the ES was activated before the AD in all conditions in the young participant. Conversely, the ES was activated after the AD during VRT and VART in the older participant. Nevertheless, activity of the ES is advanced to before activity of the AD during VSRT in the older participants. Group results demonstrated no effect of Condition (*F*_2,80_ = 0.52, *P* = 0.60) but an interaction of Condition × Group (*F*_2,80_ = 5.94, *P* = 0.004; η_p_^2^ = 0.13) on APAs of the ES during shoulder flexion. *Post hoc* Mann–Whitney *U* tests showed that timing of APA was delayed during VART (older: 0.90 ± 8.67 ms; young: −7.83 ± 9.20 ms; *Z* = −2.90; *P* = 0.004) in older participants, compared with young participants. However, there was no difference in the timing of APAs between groups in EMG onset of the ES with respect to EMG onset of the AD during VSRT (older: −0.02 ± 7.15 ms; young: −4.78 ± 2.22 ms; *Z* = −1.84; *P* = 0.07; Figure 5b).

**FIGURE 6:**
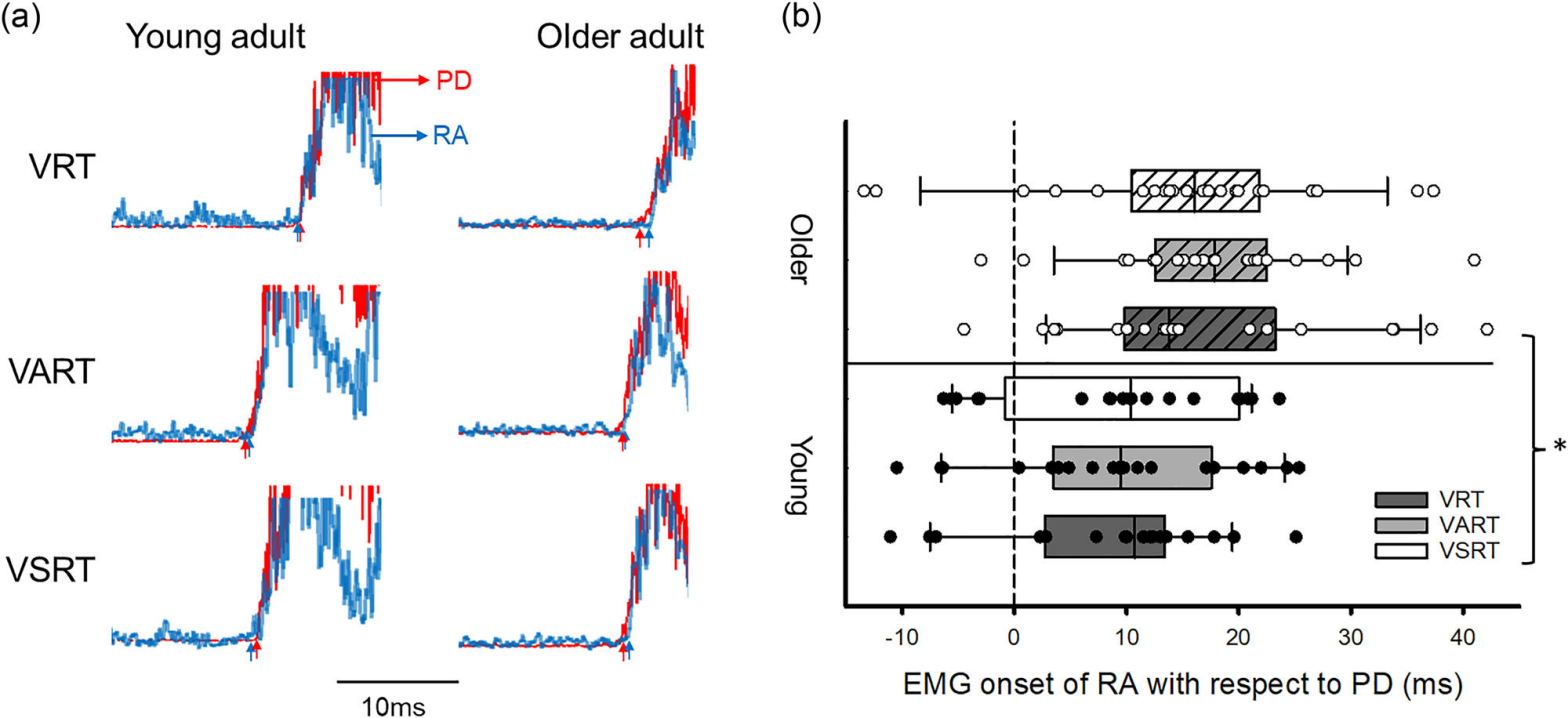
Anticipatory postural adjustments (APAs) during the shoulder extension task. (a) Rectified electromyography (EMG) traces in representative young (left) and older participants (right) during the visual, visual acoustic and visual startle reaction time conditions (VRT, VART and VSRT, respectively). EMG onset was measured during all conditions and arrows below each of the traces indicate the EMG onset in each of the conditions tested. (b) Group data (young: *n* = 20; older: *n* = 22) showing the difference in EMG onset between the rectus abdominis (RA) and posterior deltoid (PD). The abscissa shows the group. The ordinate shows the difference in EMG onset of RA to PD. Continuous lines indicate median values, and outer edges of the box represent the 25th and 75th percentiles. The upper and lower whiskers denote maximum and minimum values. Filled and open circles indicate individual data of young and older participants, respectively. *P < 0.05 comparisons between groups.

Figure 6a illustrates raw rectified EMG traces of PD and RA during VRT, VART and VSRT in a representative young participant and older participant. Note that the difference in EMG onset between PD and RA is less and the relationship of timing of activation between the two muscles remains similar across all conditions in both participants. Repeated‐measures ANOVA showed no effect of Condition (*F*_2,80_ = 0.45, *P* = 0.64) or interaction of Condition × Group (*F*_2,80_ = 0.94, *P* = 0.39) on APAs of the RA during shoulder extension. Furthermore, changes in APAs as a result of the startling stimulus to the acoustic stimulus did not correlate with ES MVCs in either young (rho = 0.11; *P* = 0.66) or older participants (rho = −0.31; *P* = 0.17).

To minimise respiration‐related activity of the RA, we controlled for the tasks being always performed during inspiration in five of the young participants (*n* = 5). Nevertheless, this did not seem to affect the results of APAs of the RA; APAs were similar across the VRT (17.06 ± 3.58 ms), VART (14.81 ± 6.67 ms) and VSRT (14.42 ± 7.85 ms). Furthermore, APAs of the ES during inspiration also followed the main results; APAs were −13.34 ± 8.56, −7.92 ± 6.31 and −4.55 ± 3.37 ms during VRT, VART and VSRT, respectively.

## 4 DISCUSSION

Our results demonstrate the contribution of the reticulospinal tract to both the prime mover, that is, shoulder muscles, and the non‐prime mover, that is, postural muscles of the trunk, during a self‐initiated postural task involving APAs of the trunk in both younger and older adults. Interestingly, the contributions of the reticulospinal tract differed in extent from the control of the ES and RA with ageing. The startling cue accelerated the onset of the ES with respect to the onset of the AD during shoulder flexion but did not change the onset of the RA with respect to the onset of the PD during shoulder extension in older adults. We suggest there is a differential effect of ageing on the reticulospinal input to the ES and RA during postural tasks, potentially reflecting the strength of the reticulospinal projections to the muscles as well as different functions of the muscles. This knowledge might help the design of exercise training for trunk muscle function and postural control in ageing.

### 4.1 Contributions of the reticulospinal tract to APAs of trunk muscles in young adults

In this study we provide evidence of the StartReact paradigm influencing the ES and RA during APAs of a self‐initiated postural task in healthy adults. We observed that EMG onset of the AD, the prime mover of shoulder flexion, and of the ES, the non‐prime mover, shortened when a startling cue was applied, suggesting the reticulospinal tract contributed to voluntary movement (Tapia et al., 2022) and there is a non‐specific influence of the reticulospinal tract on the muscles in action. Our results are supported by previous studies showing that onset of motor response in limb muscles was accelerated when a startling cue was applied in humans (Baker & Perez, 2017; Brown et al., 1991; Carlsen, 2015; Valls‐Sole et al., 1999). Additionally, our results were in keeping with other studies demonstrating shortening in movement onset in both the arm and the trunk during forward reaching (Yang et al., 2019) and rapid unilateral shoulder flexion (Tsao et al., 2009). It is suggested that the shortening in motor response is due to engagement of subcortical structures such as the reticular formation, which mediates spinal interneurons and motoneurons via the reticulospinal tract and thereby influences spinal excitability (Carlsen & Maslovat, 2019; Germann et al., 2023; Yeomans & Frankland, 1995).

Furthermore, we investigated the influence of the StartReact on APAs of the ES and RA during shoulder flexion and shoulder extension, respectively. Our young participants showed an early ES activity prior to EMG onset of the AD. Additionally, most of our young participants showed activation of PD preceding activation of the RA during shoulder extension by ∼9 ms. Nevertheless, as the EMG onset of the RA was less than 50 ms after the EMG onset of PD, activity of the RA still fell within the APA window and was still regarded as a feedforward response (Tsao et al., 2009). Prior work reported that amplitudes of motor evoked potentials (MEPs) of the ES were increasing before the onset of the AD and that the increased MEPs of the ES were accompanied by reduced intracortical inhibition (Chiou et al., 2018), suggesting a motor cortical modulation to APAs. Work has shown that when a startling stimulus preceded transcranial electrical stimulation (TES) or a cervico‐medullary electrical stimulation by 80 ms, MEP amplitudes were increased compared with the electrical stimulation delivered alone (Germann & Baker, 2021; Sangari & Perez, 2020). Additionally, a startling stimulus preceding transcranial magnetic stimulation by 50 ms suppressed corticospinal excitability at the cortical level (Tazoe & Perez, 2017). Those studies suggest that neural circuits of the primary motor cortex and reticular formation interact. Whilst we observed the shortening in EMG onset of the ES and RA during the VSRT, the relationships between ES and AD and RA and PD did not change significantly in young adults. Our results suggest that whilst startling stimuli may influence cortical and subcortical excitability, they do not seem to change preplanned motor programmes, such as APAs.

### 4.2 Contributions of the reticulospinal tract to APAs of trunk muscles in older adults

Our results showed that APA was delayed in older participants during VRT and VART, meaning that the shortening of the reaction time associated with the acoustic stimuli was uniform in both the shoulder and trunk muscles, reflecting different timing for the brain receiving auditory and visual stimulation (Kemp, 1973), regardless of age. However, APA of the ES was advanced by the startling stimuli in the older group to an extent that is the same as that for the young group, suggesting a greater reticulospinal input to the ES, and thereby an acceleration of the timing of the activity for postural control. Conversely, the same modulation from the startling stimulus was not observed in the RA in the older group; RA APA during VSRT was the same for young and older participants. An intriguing question is why does a startling cue have differential influences on APAs of the ES and RA in older adults? Our working hypothesis is that these results reflect differences in the relative strength of contributions from the reticulospinal tract to these muscles, which become more apparent when the pathways degenerate due to ageing. Research has shown that reticulospinal neurons relaying the pyramidal tract mediate longissimus lumborum motoneurons (Galea et al., 2010), suggesting that the reticulospinal input can influence corticospinal excitability. Indeed, after a corticospinal lesion such as stroke and spinal cord injury, an upregulation of the reticulospinal tract has been reported for motor recovery (Baker, 2011; Baker & Perez, 2017; Sangari & Perez, 2020; Yang et al., 2019). With ageing, corticospinal fibres degenerate and that influences corticospinal modulation of APAs. Our previous work showed that the amplitudes of ES MEPs during the APA window correlated with the onset of the ES in older adults but not in young adults (Rowland et al., 2021), suggesting an association between corticospinal excitability and APA control. A startling cue activating the reticulospinal tract and decreasing the threshold of spinal motoneuron excitability may therefore compensate for the deficiency of the corticospinal tract, thereby normalising the onset of ES with respect to the AD.

Different from the ES, anatomical studies have demonstrated that the reticular formation provides inputs to phrenic and abdominal motoneurons for respiratory function (Billig et al., 1999, 2000), in addition to postural control. It remains unclear how the reticulospinal tract modulates both respiration and postural adjustments of the RA, but this multi‐function of the reticulospinal tract to the RA could become disadvantageous when it degenerates with ageing. Nevertheless, previous work has shown that the onset of feedforward postural adjustments was faster than that of the abdominal muscles in expiration when the abdominal motoneurons were supposed to be more activated and receiving input from the reticulospinal tract (Tsao et al., 2009). Additionally, RA activity correlating with PD activity indicated that activation of the RA during the APA window was mainly associated with shoulder extension for postural control. Moreover, when controlling for the reticulospinal input to the RA for respiration, the results did not change. Taken together, it is unlikely that our results were influenced by the role of the reticulospinal tract in modulating respiration of the RA.

Our working hypothesis on the differential functional contributions from the reticulospinal tract to the RA and ES was formed based on the reticulospinal contributions to muscle strength and the decline in strength of the RA being faster than that of the ES with ageing. Studies have suggested the reticulospinal tract contributes to muscle strength (Glover & Baker, 2020; Maitland & Baker, 2021; Skarabot et al., 2022). Maximal motoneuron output was increased during a startling cue in human adults (Skarabot et al., 2022). Additionally, reticulospinal function, measured by ipsilateral MEPs elicited by TMS, correlated with hand grip strength in older adults (Maitland & Baker, 2021). Moreover, work in non‐human primates has reported adaptations of the reticulospinal pathways after resistance training (Glover & Baker, 2020). It is reported that maximal force of trunk flexors is lower than that of trunk extensors (Ben Moussa Zouita et al., 2018; El Ouaaid et al., 2013; Garcia‐Vaquero et al., 2020). Research has shown that age‐related declines in maximal strength happened earlier in trunk flexors than trunk extensors (Sasaki et al., 2018), suggesting differential age‐related adaptation in the descending motor pathways projecting to the trunk flexors and extensors. Based on those findings, we speculate that the reticulospinal tract may increase input to the ES, but not to the RA, to maintain preparatory motor control ability in older adults. Furthermore, prior work has suggested that the monoaminergic system plays a role in the efficacy of descending motor pathways (Heckmann et al., 2005; Lee & Heckman, 2000) and it has been seen to suffer from ageing (Pagano et al., 2017; Shibata et al., 2006), which could impair motor function at the level of the motoneurons (Liu et al., 2020), such as reduced motoneuronal discharge rates which could result in reduced muscle strength and functional impairments in the older participants (Hassan et al., 2021; Orssatto et al., 2023). Given the influence of the monoaminergic system on age‐related motor dysfunction, it is possible that the system may modulate APAs of the trunk by interacting with the reticulospinal tract, which requires further investigation.

It is important to discuss some aspects of the methodology used in this study that might have influenced the results. Firstly, we did not screen out trials based on startling reflexes of the SCM as suggested in previous work (Valls‐Sole et al., 1999). One reason was because the SCM is activated at low levels as a postural muscle of the neck in standing, which may decrease the specificity of the activation to the startling stimuli. We observed startling responses in the participants during the VSRT but did not always see SCM activation in the trials, in keeping with previous studies reporting the SCM activation being unreliable during startling cues (Baker & Perez, 2017; Dean & Baker, 2017). Additionally, the proportion of the reaction time that shortened during the startling cue observed in our study was comparable to what was reported in previous studies (Baker & Perez, 2017; van Lith et al., 2018; Yang et al., 2019), indicating that our StartReact paradigm activated the reticulospinal tract. Another methodological consideration is the loss of hearing with ageing. We asked our older participants to wear their hearing aids as normal during the testing, but we did not formally assess their hearing ability in this study. It could be that the desired sound level was lower to the older participants than to the young participants. We observed startling responses and effects of startling stimuli on reaction time and APAs in our older participants, suggesting the activation of the reticulospinal tract under our StartReact paradigm. Nevertheless, we suggest including a hearing test as part of the protocol when applying the StartReact paradigm in older adults. Furthermore, our participants extended the shoulders to their maximal range of motion (45–60°), whilst they flexed the shoulders from the sides to ∼90°. Although we only measured onset of the movement and did not analyse EMG activity throughout the movement in full, the extent of contributions of the reticulospinal tract to kinematics of motor tasks remains unknown. Finally, given the EMG activity of MVC and reaction time was obtained at different postures, the influence of posture on EMG activity would need to be considered when interpreting the results of EMG amplitudes.

### 4.3 Functional considerations

A systematic review reported delayed APAs in older adults compared with young adults, suggesting age‐related declines in postural control (Duarte et al., 2022). Impaired postural control is associated with balance and mobility, leading to higher risks of falling in older individuals. Our results indicate that the reticulospinal tract contributes to motor control of APAs in the ES but to a lesser extent to APAs in the RA, in keeping with previous studies reporting the start of the decline in strength of the RA being earlier than that of the ES with ageing. These findings may suggest early intervention for prevention of age‐related declines in motor function of the RA. Additionally, our findings may be relevant to other clinical populations with impaired APAs, including stroke (Dickstein et al., 2004), spinal cord injury (Chiou & Strutton, 2020) and Parkinson's disease (Bazalgette et al., 1987). Clinically, it is more difficult for patients with neurological disorders to recover motor control of the abdominal muscles than of the trunk extensors (i.e., ES). Our findings suggest the hypothesis that a greater impairment in the abdominal muscles could be due to a lesser extent of the reticulospinal contributions to the muscles. Further investigations on this hypothesis are warranted.

### 4.4 Conclusions

Our results demonstrate the influence of startling cues on both prime movers and non‐prime movers during a self‐initiated, rapid bilateral shoulder movement in young and older adults. We further reveal that the age‐related changes in the reticulospinal contributions to APAs are different between the ES and RA. Our working hypothesis is that these results reflect differences in the relative strength of functional contributions from the reticulospinal tract to these muscles, which become more apparent when the pathways degenerate due to ageing.

## AUTHOR CONTRIBUTIONS

This research was undertaken in the laboratories of The School of Sport, Exercise & Rehabilitation Sciences at The University of Birmingham, UK. Shin‐Yi Chiou, Catherine Unwin and Alice Lilley contributed to the conception and design of the experiment. Catherine Unwin and Alice Lilley performed the data collection and analysis. Shin‐Yi Chiou drafted the manuscript. Shin‐Yi Chiou, Catherine Unwin and Alice Lilley revised the manuscript critically for important intellectual content. All authors have read and approved the final manuscript and agree to be accountable for all aspects of the work in ensuring that questions related to the accuracy or integrity of any part of the work are appropriately investigated and resolved. All persons designated as authors qualify for authorship, and all those who qualify for authorship are listed.

## CONFLICT OF INTEREST

The authors declare that they have no competing interests.

## FUNDING INFORMATION

This work was not supported by external funding.

## Data Availability

The datasets generated and/or analysed during the current study are available from the corresponding author on request.
